# Sedation and Endoscopy-Assisted High-Resolution Manometry (SEA-HRM) in Patients Who Previously Failed Standard Esophageal Manometry

**DOI:** 10.3390/diagnostics14192232

**Published:** 2024-10-06

**Authors:** Daniel L. Cohen, Eyal Avivi, Sergei Vosko, Vered Richter, Haim Shirin, Anton Bermont

**Affiliations:** 1 The Gonczarowski Family Institute of Gastroenterology and Liver Diseases, Shamir (Assaf Harofeh) Medical Center, Zerifin 70300, Israel; eyalavivi4@gmail.com (E.A.); sergeivosko@gmail.com (S.V.); richterv@gmail.com (V.R.); haimsh@shamir.gov.il (H.S.); bermont@doctor.com (A.B.); 2 The Faculty of Medical and Health Sciences, Tel Aviv University, Tel Aviv 61390, Israel

**Keywords:** manometry, endoscopy, sedation, esophageal motility disorders, achalasia

## Abstract

**Objectives:** Esophageal high-resolution manometry (HRM) is the gold standard for diagnosing esophageal motility disorders, but it may be poorly tolerated and unsuccessful. We sought to evaluate a protocol for sedation and endoscopy-assisted (SEA) HRM in patients who previously failed standard HRM and assess patient perspectives towards it. **Methods:** Adult patients who previously failed HRM were prospectively enrolled. Under propofol sedation, an upper endoscopy was performed during which the HRM catheter was advanced under endoscopic visualization. If the catheter did not reach the stomach on its own, the endoscope itself or a snare was used to help it traverse the esophagogastric junction (EGJ). **Results:** Thirty patients participated (mean age 67.8, 70% female). The technical success of SEA-HRM was 100%. Twenty-two (73.3%) were diagnosed with a motility disorder including thirteen (43.3%) with achalasia. Eighteen (60%) had previously failed HRM due to discomfort/intolerance, while twelve (40%) failed due to catheter coiling in the esophagus. Subjects in the coiling group were more likely to need endoscopic assistance to traverse the EGJ (91.7% vs. 27.7%, *p* = 0.001) and have a motility disorder (100.0% vs. 55.6%, *p* = 0.010), including achalasia (75.0% vs. 22.2%, *p* = 0.004), compared to the discomfort/intolerance group. All patients preferred SEA-HRM and rated it higher than standard HRM (9.5 ± 1.3 vs. 1.9 ± 2.1, *p* = <0.001, on a scale of 1–10). **Conclusions:** SEA-HRM is a highly successful and well-tolerated option in patients who previously failed standard HRM. This should be the recommended approach in cases of failed HRM rather than secondary tests of esophageal motility.

## 1. Introduction

Esophageal high-resolution manometry (HRM) is the gold standard for diagnosing esophageal motility disorders [1]. To perform HRM, the manometry catheter is inserted via the patient’s nose and passed through the esophagus until the catheter tip reaches the stomach. As this is performed without sedation, this may be uncomfortable and not tolerated by patients who are accustomed to sedation during other gastrointestinal procedures [2]. Additionally, if there are esophageal anatomical abnormalities or a hypertensive lower esophageal sphincter, the catheter may not successfully reach the stomach. Prior studies have shown that between 2.0–13.2% of HRM studies are failed [3,4,5,6].

When HRM is unsuccessful, several options exist to evaluate esophageal motility, including performing a barium esophogram or functional luminal imaging probe (FLIP) [7]. However, both esophagography and FLIP are regarded as adjunctive tests and are not the gold standard. Therefore, a better option may be to perform HRM during endoscopy. Thus, sedation can be provided to help with patient discomfort, while endoscopic guidance can be used to aid the catheter in reaching the stomach. 

Traditionally, this approach of sedation and endoscopy-assisted (SEA) HRM was not recommended for fear that the anesthesia given to the patient would affect esophageal motility. However, a recent study has shown that the use of anesthesia has a negligible effect on HRM findings [8]. Additionally, several retrospective case series have been published using sedation and endoscopy to aid patients who previously failed HRM [9,10,11,12,13]. This suggests that SEA-HRM may be a promising alternative for patients who are unable to successfully undergo HRM.

While reports of SEA-HRM appear to be more common and it may be occasionally practiced at certain medical centers, no set protocol has ever been established, nor have any prospective studies been performed. We therefore aimed to prospectively assess the technical success of a protocol of SEA-HRM in patients who previously failed traditional HRM. Secondarily, we sought to assess which supplemental endoscopic techniques are necessary to perform SEA-HRM and how those are related to the reason why standard HRM failed. Finally, we evaluated patients’ perspectives towards SEA-HRM in comparison to standard HRM. 

## 2. Materials and Methods

### 2.1. Study Design

This was a prospective study performed between January 2022 and October 2023. It was approved by the center’s Institutional Review Board (ASF-0241-21) and all subjects provided written informed consent. The study was capped at 30 participants as this was deemed sufficient to determine the technical success rate of the SEA-HRM protocol by the Institutional Review Board.

Adult patients (age 18 or older) were eligible for the study if they had a clear indication for HRM and a prior attempted HRM study was unsuccessful, whether the prior attempt was performed at our institution or another. No sedation was provided in the standard HRM attempt other than the application of topical lidocaine gel to the catheter. All failed HRM procedures at our institution had been attempted by a senior gastroenterologist with training in neurogastroenterology. For failed HRM studies from other institutions, it was not clear who attempted to perform the study (i.e., gastroenterologist, nurse, or technician). 

The reason for the prior HRM failure was categorized as either (1) intolerance due to discomfort when attempting to pass the catheter through the nose/throat, or (2) coiling of the catheter in the esophagus with inability to pass the esophagogastric junction (EGJ). For subjects who had previously undergone attempted HRM at an outside institution, the reason for failure was taken from the procedure report or the treating gastroenterologist’s notes. 

### 2.2. SEA-HRM Protocol

The HRM system (ManoScan, Medtronic, Minneapolis, MN, USA) was prepared and calibrated in the endoscopy room. Propofol sedation was provided by the endoscopist. Propofol was chosen given its short half-life and ability to provide adequate sedation by itself.

All subjects underwent the same SEA-HRM protocol which can be seen in Supplemental Video S1. After sedation, an upper endoscopy was performed using a standard upper endoscope (Pentax Medical i10 series, Tokyo, Japan) with CO_2_ insufflation. No nasal or pharyngeal anesthesia was given. The endoscope was then pulled back into the pharynx while a second physician inserted the lubricated HRM catheter transnasally. The catheter was visualized passing the upper esophageal sphincter and entering the esophagus. If the catheter was heading towards the vocal cords, then the endoscope was used to nudge the catheter into the esophagus.

The endoscope then followed the catheter as it was advanced down the esophagus. Upon reaching the distal esophagus, the endoscope visualized if the catheter advanced on its own through the EGJ. If the catheter failed to pass through the EGJ on its own, then the endoscope was used to direct the catheter tip towards the EGJ, and the endoscope was used to push the catheter through the EGJ into the stomach. If this too failed, then an endoscopic snare was used to grab the catheter tip and advance the catheter into the stomach along with the endoscope.

Once the catheter was in the stomach, the endoscope was used to suction air from the stomach and was then carefully removed while the catheter tip remained in the stomach. If the catheter began coming back with the endoscope, then the endoscope was gently “jiggled” to allow it to return into the esophagus while the catheter remained in the stomach. The catheter was confirmed to remain in the stomach while the endoscope was in the esophagus, and this was also verified manometrically. The endoscope was then fully removed from the patient, the catheter was secured with tape to the patient, and the patient was allowed time to awaken. The patient was considered adequately awake only when they were fully conscious and able to answer questions (Aldrete score of 2 for consciousness) [14].

The standard HRM protocol was then performed according to the Chicago classification, version 4.0 (CCv4.0), while in the endoscopy suite [1]. The endoscopy bed was adjusted into the upright and supine positions as needed. The time from the end of the endoscopy until the first wet swallow was recorded. Finally, the HRM study was interpreted by an expert in neurogastroenterology according to the standard protocol [1].

### 2.3. Patient Perspectives

**Figure 1:**
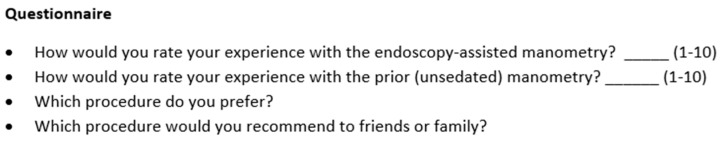
The brief questionnaire answered by subjects after completing the SEA-HRM protocol.

To assess patient perspectives on SEA-HRM, subjects were given a brief four-question questionnaire after completing the SEA-HRM protocol (Figure 1). Patients were asked to rate their experience with both SEA-HRM and traditional HRM on a scale from 1 (difficult) to 10 (easy). Additionally, they were asked which of the two procedures they preferred, and which of the procedures they would recommend to friends or family.

### 2.4. Statistical Analyses

Statistical analysis was performed using SPSS version 26 for Windows (IBM SPSS Statistics, Armonk, NY, USA). Continuous variables were reported as means with standard deviations as they were normally distributed. For comparing categorical variables, Pearson χ^2^ test was used. However, for cases in which the number of variables was low, the Fisher exact test was used. For continuous variables, the Mann–Whitney U test was performed. All statistical tests were 2-sided with *p* < 0.05 considered significant.

## 3. Results

### 3.1. Study Participants

**Figure 2:**
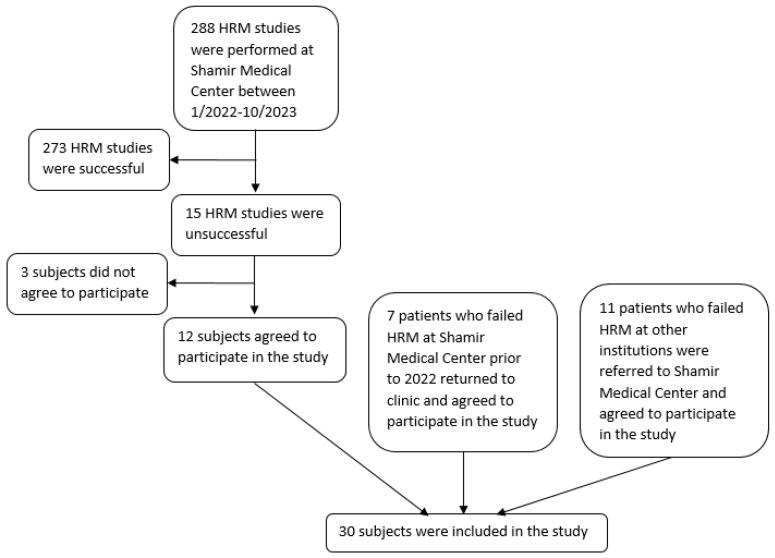
Flowchart of sources of study subjects.

During the study period, 288 HRM studies were performed in our center. Of those, 15 (5.2%) were unsuccessful, and 12 of those patients agreed to participate in this study. Additionally, seven patients who had previously had an unsuccessful HRM at our center prior to the study period returned in follow-up to our clinic and were entered into the study. Finally, 11 patients who had failed HRM at other institutions and were referred to our medical center were included (Figure 2).

Thus, a total of 30 patients (21 women, 70%) participated in the study. The mean age was 67.8 (range 21–90) with 13 (43.3%) being age 75 or older. Demographic and clinical details of the study population can be seen in Table 1.

Of the 30 subjects, 18 (60.0%) had previously failed HRM due to discomfort/intolerance, while 12 (40.0%) had failed due to coiling of the catheter in the esophagus.

### 3.2. Technical Success of SEA-HRM, the Need for Endoscopic Assistance, and Motility Diagnoses

All SEA-HRM studies (30 of 30, 100%) were technically successful. There were no adverse events. The mean dose of propofol was 155 ± 114 mg. The mean wait time between the completion of the upper endoscopy and the first wet swallow of the HRM was 6.1 ± 3.5 min. 

In 14 cases (46.7%), the catheter was advanced into the stomach without requiring assistance from the endoscope. However, in 11 cases (36.7%), the endoscope was used to push the catheter through the EGJ, while in another 5 cases (16.7%) an endoscopic snare had to be used to advance the catheter past the EGJ. 

**Figure 3:**
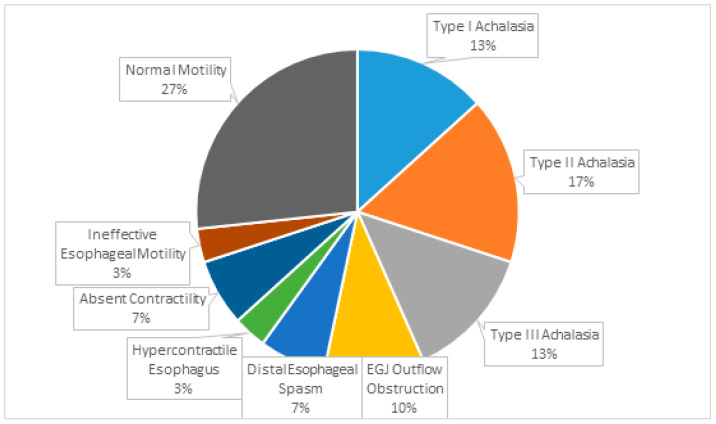
Results of the SEA-HRM studies (esophageal motility diagnoses).

Motility disorders were diagnosed in 22 (73.3%) SEA-HRM studies (see Figure 3). More than half the subjects had a Disorder of EGJ Outflow, including achalasia in 13 patients (43.3% of the overall cohort) and EGJ Outflow Obstruction in 3 (10.0%).

Comparisons between the discomfort/intolerance group and the coiling group can be seen in Table 2. Subjects in the coiling group were more likely to need endoscopic assistance to traverse the EGJ (91.7% vs. 27.7%, *p* = 0.001) and have a motility disorder diagnosed (100.0% vs. 55.6%, *p* = 0.010), including achalasia (75.0% vs. 22.2%, *p* = 0.004).

Comparisons were also performed between cases that required endoscopic assistance to pass the EGJ and those that did not (Table 3). Patients who required endoscopic assistance were more likely to have a motility disorder (100.0% vs. 42.9%, *p* = 0.001), including achalasia (68.7% vs. 14.3%, *p* = 0.003). 

### 3.3. Patient Perspectives towards SEA-HRM and Subsequent Treatments

**Figure 4:**
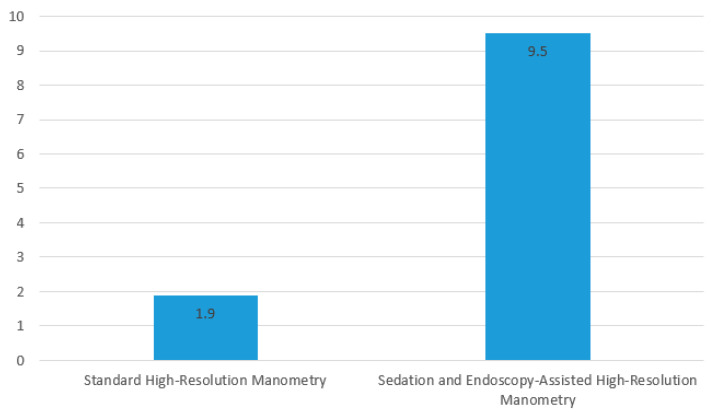
Patient satisfaction ratings of their experiences with standard HRM versus SEA-HRM (on a scale of 1 to 10).

SEA-HRM was very well tolerated. All patients (30 of 30, 100%) preferred the SEA-HRM protocol to standard HRM. Overall, the mean score for standard HRM was 1.9 ± 2.1 and increased to 9.5 ± 1.3 out of 10 for SEA-HRM (*p* ≤ 0.001, Figure 4). This higher level of satisfaction was seen in both the discomfort/intolerance group (1.2 ± 0.7 to 9.3 ± 1.6) and the coiling group (2.8 ± 2.9 to 9.7 ± 0.6), with no difference in the change in the level of satisfaction between the groups (*p* = 0.185). All subjects reported that they would recommend SEA-HRM to friends or family over standard HRM.

SEA-HRM studies led to treatment in many patients. Of the thirteen achalasia patients, nine underwent peroral endoscopic myotomy (POEM), two are currently awaiting POEM, one underwent laparoscopic Heller myotomy, and one refused treatment. Additionally, one patient with Distal Esophageal Spasm was started on a calcium-channel blocker. Finally, after normal SEA-HRM studies ruled out a motility disorder, a patient with eosinophilic esophagitis began treatment with oral budesonide and a patient with a Schatzki ring underwent endoscopic dilation.

## 4. Discussion

This is the first study to prospectively evaluate a protocol of SEA-HRM in patients who previously failed standard HRM. We found that this protocol is highly effective, leads to a high yield of motility disorders, and is very well tolerated.

While HRM is the gold standard for diagnosing esophageal motility disorder, it remains an uncomfortable test for patients [2]. The catheter needs to be inserted transnasally, and as this is done without sedation, this may be poorly tolerated by patients who are often accustomed to sedation during other procedures. Plus, the catheter needs to be advanced until it reaches stomach which may be difficult if there is an underlying esophageal disease, whether it be anatomic or due to dysmotility. Thus, the presence of achalasia, a large hiatal hernia, and an epiphrenic diverticulum have all been identified as risk factors for a failed HRM study [3,4,15].

When HRM fails, one option that is becoming more popular is to perform FLIP since it is performed with sedation and can assess elements of esophageal motility [7]. However, while there is growing literature on its uses, it still remains an adjunctive test for evaluating and diagnosing esophageal motility disorders such as achalasia. Thus, we sought to show that by using the advantages of the world of endoscopy, we could aid patients in the realm of neurogastroenterology to undergo the gold standard test to diagnose esophageal motility disorders. We performed SEA-HRM on 30 patients who had previously failed HRM and achieved 100% technical success. This included many elderly patients (43.3% were older than age 75) and many who had an underlying motility disorder (73.3% of the cohort).

SEA-HRM was well tolerated. Upon questioning after the procedure, all patients preferred it compared to standard HRM and rated it much higher. While this study only evaluated patients who previously failed HRM, this suggests that there may be patients who would prefer it over standard HRM even without first failing standard HRM. Thus, patients who are highly sensitive or too nervous to attempt an unsedated procedure may benefit from upfront SEA-HRM. It may also be worth considering performing upfront SEA-HRM in patients who have the known risk factors for failing standard HRM, such as those with achalasia, a large hiatal hernia, or an epiphrenic diverticulum, without first having to fail HRM [3,4,15].

We also identified differences between those who had previously failed HRM due to discomfort/intolerance versus those with catheter coiling in the esophagus. The coiling group was significantly more likely to require endoscopic assistance and have achalasia, as has been shown in other studies [3,4]. However, there still were cases of achalasia in the discomfort/intolerance group. This shows that patients who failed HRM due to discomfort/intolerance cannot be assumed to not have achalasia and should certainly undergo evaluation for a motility disorder with SEA-HRM. Since most subjects in the discomfort/intolerance group did not require endoscopic assistance, it is possible that they could have tolerated HRM with only sedation being given. This was not attempted in our study, but it may be worth evaluating the possibility of sedation-assisted HRM (without endoscopy) in this group in a future study. The use of a nasopharyngeal airway has also been shown to be beneficial in these patients [16]. Unsedated endoscopy may also have been successful in some patients who only needed assistance in traversing the EGJ, but it is not an option in our locale.

Some concerns about the use of SEA-HRM include the effect of anesthesia or gastric insufflation on HRM measurements, longer procedure times, added financial costs of the procedure, and possible complications from the endoscopy or sedation [2]. The study from Su et al. showed minimal effect on HRM findings due to sedation, although midazolam and fentanyl were used in that study [8]. We used propofol in this study due to its short half-life. As our mean wait time was only 6.1 min, this appears to alleviate any fears of long procedure times. Our wait time was also similar to a retrospective case series using propofol sedation [9]. Finally, there were no complications in our study, as well as in two retrospective case series, showing that SEA-HRM is safe [9,10]. 

There are some limitations to our study. It was a single-center study so its results may not be generalizable. It included only 30 subjects, but as all of these SEA-HRM studies were successful, it was not felt that adding additional subjects was necessary as the technical success was clearly proven. We used propofol mono-sedation given its short half-life and ease of use, but this may not be available at all institutions and may require an anesthesiologist, nor has the effect of propofol on HRM findings been evaluated. The effect of gastric insufflation on measurements of gastric pressure and integrated relaxation pressure has also not been evaluated. In an attempt to avoid residual gastric distension, we used CO2 for insufflation, which more quickly leaves the gastrointestinal tract than air, and suctioned gastric air via the endoscope prior to removing it, but it remains uncertain if this could affect the results of HRM testing. As a solid-state catheter was used, we cannot be certain that the same SEA-HRM technique will work in water-perfused systems which may be more fragile. Finally, we did not calculate the cost of the procedure, but requiring two gastroenterologists, a possible anesthesiologist, and an upper endoscopy certainly add to the financial costs of SEA-HRM versus standard HRM. However, given the high costs of alternative procedures such as FLIP for cases of failed HRM, the potential costs of SEA-HRM may not be very different [7]. These cost-related issues require further study.

In conclusion, we have shown that a protocol of SEA-HRM utilizing propofol is highly successful and well-tolerated by patients who previously failed standard HRM. We believe that performing SEA-HRM should be the recommended approach in patients who fail standard HRM, allowing for the gold standard for diagnosing motility disorders (HRM) to be performed instead of an adjunctive test such as FLIP. Consideration should also be given to performing upfront SEA-HRM in those at a high risk of failing standard HRM or those refusing unsedated HRM.

## Figures and Tables

**Table 1:** Details of the study population.

	n = 30
**Demographics**	
Age (years)	67.8 ± 16.8
Age ≥ 75	13 (43.4%)
Age < 75	17 (56.7%)
Gender	
Male	9 (30.0%)
Female	21 (70.0%)
**Medical history**	
Body Mass Index	26.2 ± 6.3
Ischemic Heart Disease	3 (10.0%)
Cerebral Vascular Accident	4 (13.3%)
Hypothyroidism	4 (13.3%)
Scleroderma	1 (3.3%)
Diabetes	3 (10.0%)
Alcohol abuse	0 (0.0%)
Current smoker	6 (20.0%)
Opioid use	3 (10.0%)
Prior bariatric surgery	3 (10.0%)
Prior fundoplication	1 (3.3%)
Epiphrenic diverticulum	2 (6.7%)
Eosinophilic esophagitis	1 (3.3%)
Barrett’s esophagus	1 (3.3%)
Schatzki ring	1 (3.3%)
**Primary indication for HRM**	
Dysphagia	23 (76.7%)
Vomiting/regurgitation	5 (16.7%)
Chest pain	2 (6.7%)
**Reason prior HRM failed**	
Patient intolerance	18 (60.0%)
Coiling in esophagus	12 (40.0%)

**Table 2:** Comparison between those who failed HRM due to intolerance and those with catheter coiling.

	Discomfort/Intolerance	Catheter Coiling	
	n = 18	n = 12	*p*-Value
**Demographics**			
Age (years)	65.6 ± 17.1	71.2 ± 16.3	0.285
Age ≥ 75	12 (66.7%)	5 (41.7%)	0.176
Age < 75	6 (33.3%)	7 (58.3%)	
Gender			0.102
Male	3 (16.7%)	6 (50.0%)	
Female	15 (83.3%)	6 (50.0%)	
**SEA-HRM**			
Propofol dose (mg)	151.7 ± 110.7	161.7 ± 123.5	0.950
Endoscope push	4 (22.2%)	7 (58.3%)	0.063
Endoscopic snare use	1 (5.6%)	4 (33.3%)	0.128
Endoscopic assistance (push or snare)	5 (27.7%)	11 (91.7%)	0.001
Technical success	18 (100.0%)	12 (100.0%)	–
**Motility diagnoses**			
Normal motility	8 (44.4%)	0 (0.0%)	0.010
Motility disorder	10 (55.6%)	12 (100.0%)	0.010
Achalasia	4 (22.2%)	9 (75.0%)	0.004

**Table 3:** Comparison between those who required endoscopic assistance during SEA-HRM and those who did not.

	Endoscopic Assistance	No Endoscopic Assistance Needed	
	n = 16	n = 14	*p*-Value
**Demographics**			
Age (years)	70.2 ± 15.4	65.1 ± 18.4	0.473
Age ≥ 75	8 (50.0%)	5 (35.7%)	0.431
Age < 75	8 (50.0%)	9 (64.3%)	
Gender			0.118
Male	7 (43.7%)	2 (14.3%)	
Female	9 (56.2%)	12 (85.7%)	
**SEA-HRM**			
Propofol dose (mg)	176.2 ± 130.6	132.1 ± 90.6	0.400
Technical success	16 (100.0%)	14 (100.0%)	–
**Motility diagnoses**			
Normal motility	0 (0.0%)	8 (57.1%)	0.001
Motility disorder	16 (100.0%)	6 (42.9%)	0.001
Achalasia	11 (68.7%)	2 (14.3%)	0.003

## Data Availability

Data are available from the corresponding author upon reasonable request.

## Supplementary Materials

The following supporting information can be downloaded at: https://www.mdpi.com/article/10.3390/diagnostics14192232/s1, Video S1: The SEA-HRM protocol. After sedation, a standard upper endoscopy is performed. The endoscope is then pulled back into the pharynx while a second physician inserts the HRM catheter transnasally. The catheter is visualized entering the esophagus. The endoscope then follows the catheter down the esophagus and into the stomach. If the catheter fails to pass through the EGJ on its own, then the endoscope is used to push the catheter through the EGJ into the stomach. If this too fails, then an endoscopic snare is used to grab the catheter tip and advance the catheter into the stomach along with the endoscope. Once the catheter is in the stomach, the endoscope is carefully removed.
